# Gratitude and Problem Behaviors in Adolescents: The Mediating Roles of Positive and Negative Coping Styles

**DOI:** 10.3389/fpsyg.2019.01547

**Published:** 2019-07-16

**Authors:** Peizhen Sun, Yudi Sun, Hongyan Jiang, Ru Jia, Zhiyuan Li

**Affiliations:** ^1^School of Education Science, Jiangsu Normal University, Xuzhou, China; ^2^School of Management, China University of Mining and Technology, Xuzhou, China; ^3^Division of Primary Care, School of Medicine, University of Nottingham, Nottingham, United Kingdom

**Keywords:** gratitude, positive coping style, negative coping style, internalizing behavior, externalizing problem behavior

## Abstract

The current study investigated the relationship between gratitude, internalizing and externalizing problem behaviors, along with the mediating roles of positive and negative coping styles therein. A sample of 589 Chinese adolescents completed the Gratitude Questionnaire (GQ-6), the Simplified Coping Style Questionnaire (SCSQ), the Irritability, Depression, and Anxiety Scale (IDAS), and the Aggression Questionnaire (AQ). Results of structural equation modeling showed that (1) the total effects of gratitude on both internalizing and externalizing problem behaviors were all significant and (2) both positive and negative coping styles mediated the links between gratitude and two types of problem behaviors. Thus, cultivating gratitude and developing adaptive coping style may help adolescents rectify problem behaviors.

## Introduction

Adolescence is the critical stage of individual’s physical and mental development. Individuals at this stage are more likely to suffer from problem behaviors, which can carry on to adulthood, developing further into life-long problems (Connell et al., 2009; Huang et al., 2012). Problem behavior refers to abnormal behaviors that are harmful to individuals’ physical and mental health, which can be divided into internalizing problem behavior and externalizing problem behavior (Owens and Hoza, 2003).

Internalizing problem behaviors refer to those emotion-oriented problem behaviors, connoting the negative emotions experienced by individuals, such as anxiety, depression, and withdrawal (Eisenberg et al., 2001). By contrast, externalizing problem behaviors refer to those behavior-oriented problem behaviors, encompassing individuals’ non-adaptive behaviors violating social norms and even the law (Colder et al., 2017). For instance, aggression is a common externalizing problem behavior among adolescents, which can be defined as the behavior by one person intended to harm another person (Dodge et al., 2006). Delinquency is also an externalizing problem behavior, which is defined as the behavior by one individual that violates the formal norms and it even makes the person subject to the court (Shaw and Gross, 2008).

The problem behaviors could not only be detrimental to the physical and psychological health of adolescents themselves, but also inflict damage on others even the whole society (Goldstein et al., 2005; Liang et al., 2018). Understanding possible contributors of problem behaviors among adolescents will help reduce the occurrence of such behaviors and resolve the issues these behaviors might bring. Therefore, this study attempts to examine the effect of gratitude on problem behaviors among adolescents. Furthermore, this study aims to expand previous literature by probing the underlying mediating mechanism therein in the Chinese context. Based on previous evidences (Yu et al., 2011; Lin, 2015; Deng et al., 2016; Liang et al., 2018), we propose the hypotheses that gratitude may exert significant influence on both internalizing problem behavior and externalizing problem behavior, and both positive and negative coping style will play mediating roles in the links between gratitude and problem behaviors.

### Gratitude and Problem Behavior

One of the possible contributors of problem behavior is gratitude (Wood et al., 2008; Petrocchi and Couyoumdjian, 2015). Gratitude can be defined as the generalized tendency of a person in experiencing and responding with grateful emotions to others’ kindness (McCullough et al., 2002). Evidence suggested that gratitude was negatively connected with internalizing problem behavior. For example, Wood et al.’s (2008) longitudinal studies, using a full cross-lagged panel design, demonstrated that gratitude could directly protect people from depression and anxiety, which were the observable indicators of internalizing problem behavior (Petrocchi and Couyoumdjian, 2015). Along the same line, Liang et al. (2018) extended this relationship in different context by re-verifying the significant effect of gratitude on depression in Chinese culture.

Prior studies have also probed the vital role of gratitude in externalizing problem behaviors (DeWall et al., 2012; Deng et al., 2016). For instance, DeWall et al. (2012) implemented a study using multi-approaches such as cross-sectional design, longitudinal design, and experimental method and discovered that gratitude was negatively related to externalizing problem behavior like aggression. Similarly, Deng et al. (2016) observed a significant decrease in levels of aggression behavior among violent criminals after a gratitude-based intervention.

Apart from these empirical findings, Bono and Froh (2009) proposed the Intrinsic and Extrinsic Goal Theory, which can illustrate the significant effects of gratitude on both internalizing and externalizing problems. According to this theory, gratitude can promote the pursuit of individual’s intrinsic goals and reduce the pursuit of extrinsic or materialistic goals. Specifically, compared to extrinsic goal pursuers who crave for wealth, fame, and reputation, intrinsic goal pursuers are more concerned with establishing good interpersonal relationships and achieving personal growth (Froh et al., 2011). The latter can better meet basic psychological needs of relatedness, competence, and autonomy. Moreover, individuals who are satisfied with basic psychological needs can achieve better positive development outcome and inhibit internalizing and externalizing problem behaviors (Bono and Froh, 2009). Therefore, based on the available empirical evidences and the Intrinsic and Extrinsic Goal Theory, the first hypothesis was proposed as follows:

*Hypothesis 1*: Gratitude will be negatively associated with both internalizing and externalizing problem behaviors.

### The Role of Coping Style in Gratitude and Problem Behavior

Despite that the link between gratitude and problem behavior seems to be well established, the inner mechanisms underlying this relationship have been less studied. Specifically, there is little research into the roles of individual cognitive or behavioral strategies in the relationship between gratitude and problem behavior (DeWall et al., 2012). Yet, individual cognitive and behavioral strategies, for instance, coping styles, may be closely related to both gratitude and problem behaviors (Fan and Chen, 2007; Lin and Yeh, 2014; Whitman and Gottdiener, 2015).

#### Gratitude and Coping Style

Coping style is regarded as a cognitive or behavioral strategy adopted by individuals when facing stressful situations (Thomas et al., 2011; Lin and Yeh, 2014). It can be divided into two types: positive coping style (e.g., positive reappraisal, problem-focused coping, creation of positive meaning, and growth) and negative coping style (e.g., self-blame, withholding, and escape; Folkman and Lazarus, 1986; Folkman and Moskowitz, 2000). Fredrickson and Joiner (2002) pointed out that positive emotions were significantly correlated with coping styles. Fredrickson (2004) further indicated that as one of positive emotional traits, gratitude can optimize individuals’ coping styles, enhancing individuals’ ability to cope with stress and adversity. Furthermore, Wood et al. (2007, 2010) revealed significant associations of gratitude and different coping styles. They suggested that gratitude was positively correlated with positive coping styles (e.g., seeking emotional and instrumental social support, planning or positive reinterpreting the situation, and finding the potential for growth) and negatively linked to negative coping styles such as behavior disorder, self-accusation, rejection, and substance abuse. Drawing on these arguments, the following hypothesizes would be tested:

*Hypothesis 2*: Gratitude will be significantly associated with coping style.

*Hypothesis 2a*: Gratitude will be positively connected with positive coping style.

*Hypothesis 2b*: Gratitude will be negatively connected with negative coping style.

#### Coping Style and Problem Behaviors

Several studies have demonstrated that coping style could significantly predict problem behavior (Folkman and Lazarus, 1986; Fan and Chen, 2007; Howerton and Van Gundy, 2009). Specifically, negative coping style is deemed as maladaptive and can elicit both externalizing and internalizing problem behaviors in the long run, conversely, positive coping style can serve as a buffer to impede problem behavior (Noh and Kaspar, 2003; Scarpa and Haden, 2006; Whitman and Gottdiener, 2015). From the perspective of externalizing problem behavior, Scarpa and Haden (2006) asserted that adopting negative coping styles (i.e., disengagement, avoidant strategies, or emotion-focused strategy) may increase aggression scores, while positive coping style (i.e., problem-focused) could mitigate the risk of aggression or even prevent it. Similarly, Whitman and Gottdiener (2015) tested the findings in a cross-sectional study that maladaptive coping styles can predict higher level of aggression, while adaptive or positive coping styles produce the opposite outcome.

Furthermore, coping style has been proven to be a significant factor in internalizing problem behavior. Folkman and Lazarus (1986) found that individuals were more likely to be diagnosed with depression in the future when they were accustomed to adopt negative coping styles, such as refusal, withdrawal, avoidance, distraction, and fantasy. These findings are not only applicable to western culture. Noh and Kaspar’s (2003) study on Korean immigrants in Japan also showed that positive problem-focused coping style can effectively reduce depression, while negative emotion-focused coping style may bring mental health problems. In Chinese context, Fan and Chen (2007) reached the same conclusion that passive coping styles may induce depression and anxiety, and positive coping styles may in turn neutralize them. Based on these evidence mentioned above, a series of hypothesizes were put forward as follows:

*Hypothesis 3*: Coping style will be significantly associated with problem behavior.

*Hypothesis 3a*: Positive coping style will be negatively related to both externalizing and internalizing problem behaviors.

*Hypothesis 3b*: Negative coping style will be positively related to both externalizing and internalizing problem behaviors.

#### The Broaden-and-Build Theory

Broaden-and-Build Theory (Fredrickson, 2001, 2004) may provide a theoretical explanation for the relations among gratitude, coping style and problem behaviors. Fredrickson (2001) suggested that positive emotions can broaden people’s instantaneous thought-action abilities, constantly develop, and construct stable personal resources, which are conducive to enhancing adaptive coping. She further stated that as one of the positive affective traits, gratitude can broaden the individual’s cognitive mode, break the thinking set, and construct robust personal and social resources, including coping resources (Fredrickson, 2004). In another word, grateful people could cope well. Specifically, gratitude impels individuals to reappraise or re-frame the negative events they went through more positively. Individuals with high levels of gratitude possess more abundant coping resources, use active coping styles more flexibly, think more broadly, have fewer unrealistic delusions and social withdrawal, and thus exhibit little externalizing and internalizing problem behaviors. Based on the aforementioned empirical evidences and the Broaden-and-Build Theory, the last hypothesis was as follows:

*Hypothesis 4*: Coping style will be a significant mediator in the association between gratitude and problem behaviors.

*Hypothesis 4a*: Positive coping style will play a mediating role in the relations between gratitude and problem behaviors.

*Hypothesis 4b*: Negative coping style will play a mediating role in the relations between gratitude and problem behaviors.

## Materials and Methods

### Participants

Participants in this study were 589 students (303 boys and 286 girls, aged from 12 to 15 years, mean age is 13.61 years, SD = 1.87) who were recruited using the random cluster sampling method, from three junior high schools in China.

This research was approved by the Institutional Review Board at the university of the first author. Consistent with institutional review board procedures, we first contacted the administrators in junior middle schools and got the consents for their students participating in our study. Then, the informed consents were obtained from the participants and their parents. After that, the participants filled in a packet of self-report questionnaires in the classrooms independently and anonymously.

### Measures

All the measures in this study were in Chinese. These measures were validated in Chinese samples and were found to display good psychometric properties in previous studies (e.g., Zhou et al., 2013; Yang et al., 2014; Qiu et al., 2016; Liang et al., 2018).

#### Gratitude

The Gratitude Questionnaire-Six Item Form (GQ-6; McCullough et al., 2002) was employed to assess individuals’ gratitude. The GQ-6 is scored on a 7-point Likert-type scale (1 = strongly disagree, 7 = strongly agree) with higher scores indicating stronger gratitude. This scale consisted of six items (e.g., “I have so much in life to be thankful for”), and two items (items 3 and 5) were reversely scored. The Chinese version of GQ-6 was widely used and was proved to have good internal consistency in Chinese adolescents (e.g., Lin and Yeh, 2014; Liang et al., 2018). In this study, GQ-6 also exhibited good internal consistency, and Cronbach’s α coefficient was 0.75.

#### Coping Style

The Simplified Coping Style Questionnaire (SCSQ; Xie, 1998) was used to test two types of coping style. With a total of 20 items, the SCSQ includes two factors: positive coping style (PCS) and negative coping style (NCS). PCS includes 12 items, which mainly reflects positive stress coping strategies, such as problem solving, seeking help, and reconstruction (e.g., “Talk to others about your troubles”). NCS includes 8 items, reflecting negative stress coping strategies such as avoidance and distraction (e.g., “Relieve your worries by smoking, drinking, taking medicine and eating”). Participants rated each item on a 4-point scale based on the frequency they adopt positive or negative coping styles in their daily life (0 = never, 3 = always). The scale has been proved to have good validity and reliability in Chinese populations (Yang et al., 2014). The internal consistency of the scale in this study was good for both positive coping style subscale (Cronbach’s α = 0.80) and negative coping style subscale (Cronbach’s α = 0.71).

#### Problem Behavior

As suggested by previous studies (Xing et al., 2011; Hofer et al., 2013; Colder et al., 2017), we used depression and anxiety as the indicators of internalizing problem behavior, and aggression as the indicator of externalizing problem behavior.

Internalizing problem behavior was tested by the Irritability, Depression, and Anxiety Scale (IDAS; Snaith and Taylor, 1985). The IDAS was composed of 18 items, rated on a 4-point response scale. In this study, we only used the depression subscale (five items; e.g., “I feel happy,” reverse scored) and anxiety subscale (five items; e.g., “I feel nervous”) of the IDAS. Four items in the depression subscale and two items in the anxiety subscale were scored reversely. Previous studies have reported that the Chinese version of IDAS has good reliability (Qiu et al., 2016). The reliability of the scale was acceptable for both of the depression subscale (Cronbach’s *α* = 0.71) and the anxiety subscale (Cronbach’s *α* = 0.70).

Externalizing problem behavior was measured by the Brief Version of Aggression Questionnaire (AQ; Buss and Perry, 1992; Bryant and Smith, 2001). This questionnaire consists of 12 items, measuring four underlying factors of aggression: physical aggression, verbal aggression, anger, and hostility. Sample items include “I have threatened people I know,” and “At times I feel I have gotten a raw deal out of life.” Participant rated a series of statements on a seven-point Likert scale, ranging from 1 (strongly disagree) to 7 (strongly agree). The Chinese version of AQ has been proved to have good validity and reliability in Chinese populations (Zhou et al., 2013). In this study, Cronbach’s α coefficients of total scale and subscales were acceptable (total scale: *α* = 0.82; physical aggression: *α* = 0.75; verbal aggression: *α* = 0.70; anger: *α* = 0.70; hostility: *α* = 0.77).

## Results

### Correlations Among Study Variables

Correlations coefficients were calculated among all the variables in this study. Results illustrated that gratitude was negatively correlated with both internalizing and externalizing problem behaviors. Meanwhile, gratitude showed a positive correlation with positive coping style, and a negative correlation with negative coping style. Positive coping style was negatively related to internalizing and externalizing problem behaviors. By contrast, negative coping style was positively related to two types of problem behaviors (see Table 1).

**Table 1:** Means, standard deviations, and correlation matrix for all variables.

	1	2	3	4	5	6	7	8	9
1. Gratitude	1								
2. Positive coping style	0.31^***^	1							
3. Negative coping style	−0.16^***^	−0.06	1						
4. Physical aggression	−0.27^***^	−0.17^***^	0.23^***^	1					
5. Verbal aggression	−0.14^**^	−0.09^*^	0.22^***^	0.37^***^	1				
6. Anger	−0.18^***^	−0.12^**^	0.33^***^	0.46^***^	0.56^***^	1			
7. Hostility	−0.12^**^	−0.15^***^	0.33^***^	0.25^***^	0.46^***^	0.50^***^	1		
8. Depression	−0.25^***^	−0.21^***^	0.12^**^	0.16^***^	0.12^**^	0.14^**^	0.14^**^	1	
9. Anxiety	−0.10^*^	−0.14^**^	0.22^***^	0.14^**^	0.21^***^	0.21^***^	0.42^***^	0.27^***^	1
Mean	31.72	32.98	16.25	7.54	9.74	9.68	11.98	10.52	11.79
SD	5.27	5.41	3.10	3.17	3.84	4.49	4.81	1.82	2.36

* p < .05;

** p < .01;

*** *p < .001*.

### Testing the Measurement Model (Confirmatory Factor Analysis)

The measurement model (CFA, Model 1) contained five latent variables (gratitude, positive coping style, negative coping style, internalizing problem behavior, and externalizing problem behavior) and 15 observable indicators. The indicators of the latent variables were formed using the method of item parceling, which has been proved to be a valid method to reduce the observational error in SEM (Little et al., 2002). Results indicated that the fit indices of Model 1 were good, with *χ*^2^(79) = 195.74, *p* < 0.001; RMSEA = 0.050; SRMR = 0.054; TLI = 0.90; CFI = 0.92. The loading coefficients from latent variables to their corresponding observable variables were all significant, showing that five latent variables in this study were all well represented by their corresponding indicators.

### Testing the Structural Model

#### Testing the Total Effects of Gratitude on Problem Behavior

To test the total effects of gratitude on two types of problem behaviors, we first built a model (Model 2) with only predictive variable (gratitude) and outcome variables (internalizing and externalizing problem behaviors) and without the mediating variables. This model fits well to the data, *χ*^2^(24) = 85.46, RMSEA = 0.066, SRMR = 0.067, TLI = 0.90, CFI = 0.94. The standardized path coefficients from gratitude to internalizing (*β* = −0.44, *p* < 0.001) and externalizing problem behavior (*β* = −0.28, *p* < 0.001) all reached significance. Therefore, Hypothesis 1 was supported.

#### Testing the Mediating Effects of Positive and Negative Coping Styles

Based on our hypotheses, we built a partially mediated model (Model 3) with two mediators (positive coping style and negative coping style) and two direct paths from gratitude to both internalizing and externalizing problem behaviors. The results showed that the fit indices of Model 3 were acceptable, with *χ*^2^(81) = 67.31, *p* < 0.001; RMSEA = 0.050; SRMR = 0.055; TLI = 0.90; CFI = 0.92.

**Figure 1:**
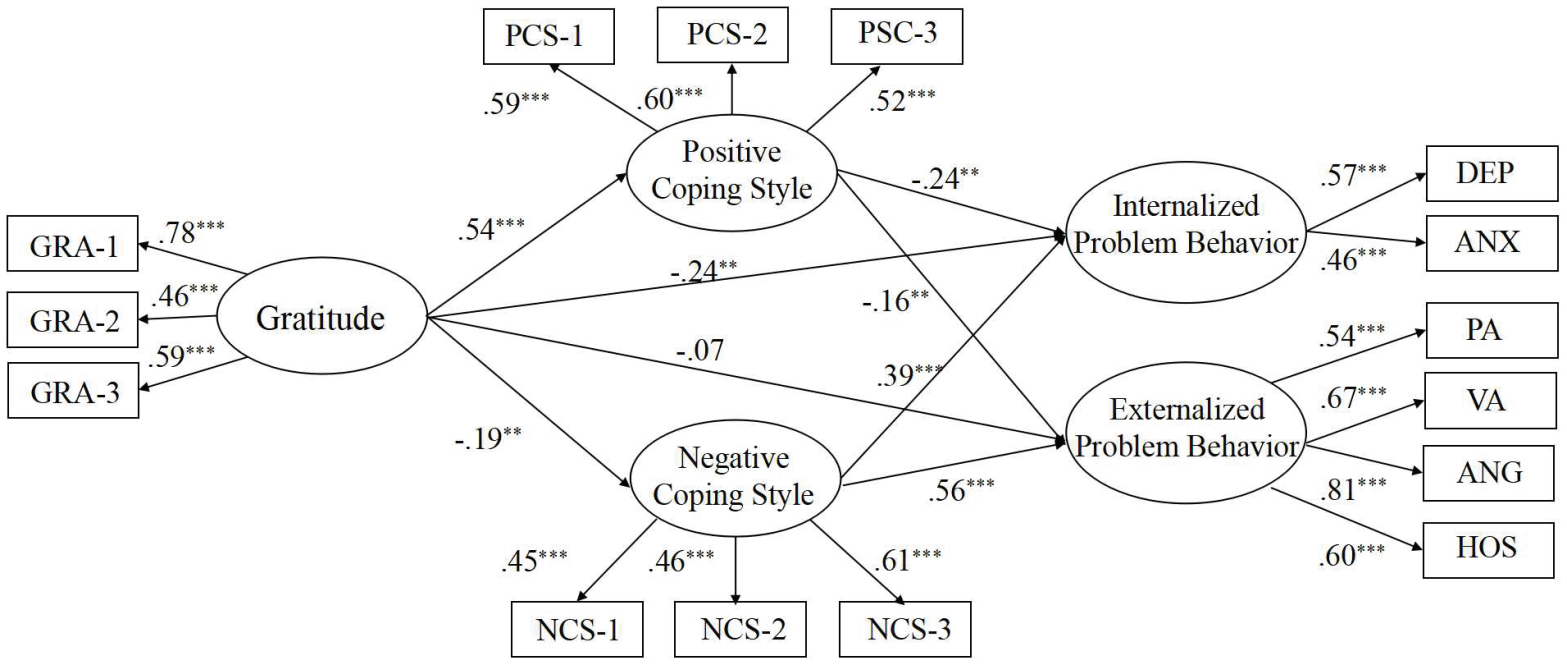
Final structural model with standardized estimates (Model 3). GRA-1, GRA-2, and GRA-3 are three parcels of gratitude; PCS-1, PCS-2, and PCS-3 are three parcels of positive coping style; NCS-1, NCS-2, and NCS-3 are three parcels of negative coping style; DEP and ANX are two dimensions of internalized problem behavior; PA, VA, ANG, and HOS are four dimensions of externalized problem behavior.

We then employed bootstrapping method to examine the significance of the effects in Model 3. The results showed that the regression coefficients from gratitude to positive coping style (*β* = 0.54, *p* < 0.001) and negative coping style (*β* = −0.19, *p* < 0.01) were all significant. Therefore, Hypothesis 2 was supported. The path coefficients from two coping styles to internalizing problem behaviors (positive coping styles: *β* = −0.24, *p* < 0.01; negative coping styles: *β* = 0.39, *p* < 0.001) and externalizing problem behaviors (positive coping styles: *β* = −0.16, *p* < 0.01; negative coping styles: *β* = 0.56, *p* < 0.001) were also significant (see Figure 1).

The bootstrapping procedures also indicated that both positive coping style and negative coping style mediated the links between gratitude and two types of problem behaviors significantly: (1) positive coping style played a mediating role in the effect of gratitude on internalizing problem behavior (indirect effect = −0.13; 95% CI: −0.21, −0.07, *p* < 0.05); (2) positive coping style played a mediating role in the effect of gratitude on externalizing problem behavior (indirect effect = −0.09; 95% CI: −0.14, −0.05, *p* < 0.05); (3) negative coping style played a mediating role in the effect of gratitude on internalizing problem behavior (indirect effect = −0.08; 95% CI: −0.13, −0.03, *p* < 0.05); (4) negative coping style played a mediating role in the effect of gratitude on externalizing problem behavior (indirect effect = −0.11; 95% CI: −0.17, −0.06, *p* < 0.05). Therefore, Hypothesis 3 was supported.

## Discussion

Adolescents’ problem behavior has become a popular research topic over years (Wood et al., 2008; Yu et al., 2011). However, the potential contributors of problem behavior and the mediating mechanisms linking these antecedent variables to problem behavior have not been systematically excavated yet (Wood et al., 2008; Watkins, 2014). For this reason, the present study aims to investigate the effect of gratitude on problem behaviors and highlighted the mediating roles of coping styles therein among Chinese adolescents.

First, the current findings contribute to the literature by revealing the effects of gratitude on both internalizing and externalizing problem behaviors, which add new evidence to existing gratitude theory (Fredrickson, 2001, 2004; Watkins, 2014). According to the Amplification Theory (Watkins, 2014), gratitude is a special cognitive resource, which can (1) enhance the positive emotions that individuals are experiencing right now, (2) regulate their emotional health and encourage self-acceptance, and (3) amplify the positive significance of individuals behind negative events, which could impede the formation of problem behaviors (Wood et al., 2008; Froh et al., 2009). On the other hand, from the perspective of Broad-and-Build Theory of Gratitude, gratitude can broaden individual’s instantaneous thought-action abilities and construct robust individual resources (Fredrickson, 2001, 2004; McCullough et al., 2008; DeSteno, 2009), including physical resources, intellectual resources, interpersonal resources, and psychological resources, which can strongly boost adolescents’ social adaptation and effectively reduce the emergence of their problem behaviors. In this study, we found that the total effects of gratitude on both externalizing and internalizing problem behaviors were all significant, which adds new evidence to existing gratitude theory and extends the study of function of gratitude to the research field of problem behaviors. Through testing the functions of gratitude in the context of Chinese culture, we have provided meaningful evidence for the external validity of previews studies.

Second, this study extends scholarly understanding of the mediating mechanism linking gratitude to problem behaviors. To the best knowledge of the authors, this is the first study that tested the mediating roles of two sorts of coping styles in the effect of gratitude on problem behaviors. As expected, in this study, we found that either positive or negative coping styles significantly mediated the effects of gratitude on problem behaviors. It means that the effects of gratitude on internalizing and externalizing problem behaviors can be achieved through the roles of positive and negative coping styles. According to the Broad-and-Build Theory of Gratitude, gratitude can broaden individual’s cognitive mode, break the thinking set, and disengage from monotonous coping styles (Fredrickson, 2001, 2004; McCullough et al., 2008; DeSteno, 2009). In other word, gratitude can inspire and motivate individuals to come up with a feasible coping strategy to obtain favorable results as soon as possible. Adolescents with high level of gratitude prefer to adopt more positive and effective coping styles to offset the harm when setbacks and frustrations occur (Wood et al., 2007, 2010), thus contributing to the acquisition of inner quietude (Watkins, 2014; Liang et al., 2018). Conversely, individuals with low level of gratitude will magnify the negative consequences of stress events and blindly adopt whichever coping strategies that come to mind in the first place, whether they are appropriate or not. Inconsiderable coping strategies will eventually fail, and the anxiety induced by such failure will in turn interfere with the individual’s choice of coping strategies, thus forming a vicious circle and immersing the individual in a negative state (Fan and Chen, 2007). In the long run, indulging in such a negative state is likely to induce symptoms of depression and anxiety internally (internalizing problem behavior) and foment physical or verbal aggression externally (externalizing problem behavior). Therefore, the finding of the significant mediation effects in this study added new proofs to existing Broad-and-Build Theory (BBT; Fredrickson, 2001, 2004) and extended the application of BBT to the research field of coping style.

The present findings also have some practical implications. First, this study found that gratitude had a significant effect on externalizing problem behaviors, which may inspire clinical psychologists and counselors to apply gratitude interventions to clinical cases where adolescents are suffering from externalizing problem behaviors. Deng et al. (2016) reported that after 5 weeks of gratitude intervention called counting blessing (Emmons and McCullough, 2003), the aggression of violent prisoners in the gratitude intervention group was significantly reduced, comparing with the control group (Deng et al., 2016). Second, the present findings provide convincing evidence of the significant effects of gratitude on internalizing problem behaviors such as depression and anxiety, which may indicate that gratitude intervention could help adolescents reducing depression and anxiety. Lyubomirsky et al. (2005) assumed that gratitude list exercise is essentially a cognitive-happiness increasing practice. Through repeated practice of positive re-memory and re-interpretation of life events, an automatic gratitude cognitive style is gradually formed to evoke positive emotions and offset negative emotions, such as depression and anxiety (Deng et al., 2016). Third, the present findings revealed convincing evidence on the significant roles of coping styles in youngsters’ problem behaviors and in the relations between gratitude and problem behaviors. These findings enlighten that behavioral interventions targeting coping styles may help individuals tackle problem behaviors. Specifically, educators and parents should encourage and guide adolescents with problem behaviors to adopt more positive coping strategies instead of negative ones. Examples of these actions include demonstrating how to convert negative perspective into positive views, offering courses to help adolescents master appropriate coping strategies, and providing more social supports.

Some limitations of the present study must be mentioned. First, this study employed the cross-sectional design; therefore, no causal relationship can be claimed. Experimental or longitudinal studies in the future are needed in order to probe the possible cause-and-effect relationships among gratitude, coping style, and problem behavior. Second, instruments of the current study were entirely based on self-report. Self-report scale is inevitably influenced by the subjectivity of participants. Therefore, more in-depth studies in the short run should combine self-rating scale with other-rating scale or supplement with physiological index, so as to offset the data bias caused by social desirability of participants. Finally, in this study, participants were recruited from China. Interpretation of the current findings may be limited in this culture context. Future prospective studies can verify whether the conclusions of the present study are consistent in different cultures.

Despite such limitations, the current study contributes to the literature by being the first to examine some psychological mechanisms, which explain the role of positive coping style and negative coping style in accounting for the relationship between gratitude and two sorts of problem behaviors. The findings in this study added new proofs to the Amplification Theory (Watkins, 2014) and Broad-and-Build Theory of Gratitude (Fredrickson, 2001, 2004), and the results aligned with previous research, that is, gratitude can reduce the emergence of the problem behaviors (Wood et al., 2008; Froh et al., 2009). Moreover, the SEM results showed that either positive or negative coping style significantly mediated the effects of gratitude on two sorts of problem behaviors. Thus, this study extends the academic understanding on the mechanism of how gratitude affects adolescents’ problem behaviors.

## Data Availability

The datasets generated for this study are available on request to the corresponding author.

## Ethics Statement

This study was carried out in accordance with the recommendations of ethics committee of Jiangsu Normal University with written informed consents from all subjects and their parents. All subjects and their parents gave written informed consents in accordance with the Declaration of Helsinki. The protocol was approved by the ethics committee of Jiangsu Normal University.

## Author Contributions

PS designed, wrote, and approved all contributions to the study. YS participated in reviewing the literature and ran all analysis for the work. HJ participated in designing the study. RJ helped to edit the manuscript. ZL participated in collecting the data.

### Conflict of Interest Statement

The authors declare that the research was conducted in the absence of any commercial or financial relationships that could be construed as a potential conflict of interest.
